# Evaluation of ATG7 and Light Chain 3 (LC3) Autophagy Genes Expression in AML Patients

**DOI:** 10.22037/ijpr.2019.1100682

**Published:** 2019

**Authors:** Mohamadreza Mohamadimaram, Mehdi Allahbakhshian Farsani, Amin Mirzaeian, Shaghayegh shahsavan, Abbass Hajifathali, Sayeh Parkhihdeh, Mohammad Hossein Mohammadi

**Affiliations:** a *Laboratory Hematology and blood Banking Department, School of Allied Medical Sciences, Shahid Beheshti University of Medical Sciences, Tehran, Iran. *; b *HSCT Research Center, Laboratory Hematology and blood Banking Department, School of Allied Medical Sciences, Shahid Beheshti University of Medical Sciences, Tehran, Iran.*; c *HSCT Research Center, Shahid Beheshti University of Medical Sciences, Tehran, Iran.*

**Keywords:** Acute Myeloid Leukemia, AML, Autophagy, ATG7, LC3

## Abstract

Background and aim: Autophagy, known as cell death type II, is a housekeeping pathway that currently has been worked on in matters of tumorigenesis and leukomogenesis. Therefore, expression levels of ATG7 and LC3 as two key genes in AML patients are targeted in this study.Material and method: This study was performed on 55 de novo AML patients against 17 healthy volunteers, acquired samples from bone marrow (BM) and peripheral blood (PB) sources in different ages and gender. The evaluation was executed by mRNA extraction, cDNA synthesis, real-time PCR and data was analyzed by SPSS. Results: Analyzed data indicate a significant decrease between expression of ATG7 and LC3 in AML patients against control (Pv < 0.05). Decrease in both genes expression was detected in most of the patients, 81.81% and 75.55%, respectively. Also LC3 overexpression was detected in 11.33% of AML patients. Moreover, a positive significant correlation between ATG7 and LC3 genes was detected (r = 0.481; Pv = 0.001). Conclusion: This study showed that significant reduction of autophagy genes in de novo AML patients is important to overcome this system and initiate leukomogenesis. It seems a new insight is required for new achievements in diagnosis, prognosis, treatment and monitoring AML patients.

## Introduction

AML is a clonal heterogenic disease of hematopoietic progenitor cells and also the most common malignant myeloid disease in adults. The average age of AML occurrence is about 70 years old. In recent years molecular biology studies have been useful in deciphering the pathogenesis of this disease. To determine the treatment response and outcome, genetic abnormalities are considered to be the most significant factors. Notwithstanding, lots of improvements have been made in curing the younger patients; conversely still patients’ condition of older age have not had such success, as overall survival of those is only few months. Such these differences originate from the comorbidity related factors, like age and the disease biology. Moreover, recent efforts in clinical studies have been focusing on assessment of target therapies. Such achievements could probably change treatment success rate. Better understanding of AML pathogenesis would result in target therapy progression intended particularly for elderly adults. Meanwhile as a matter of fact, targeting each of several types of AML genotype variants is a big challenge (1).

For decades, scientific community has been trying to understand not only the molecular mechanisms that are the basis of uncontrolled proliferation of cancer cells, but also to find how these cells are insensitive to the internal and external cell death stimuli.

Cancer cell drug resistance is mostly related to abnormally activated or defective programmed cell death (PCD), that mainly occur during apoptosis pathways; as a result, for a long time it was assumed that apoptosis reactivation could be efficient in order to improve the eradication of malignant cells. Classic apoptosis is defined by activation of caspases that account for massive protein degradation. This performance also can be fulfilled by releasing proteolytic enzymes from lysosomes (that is called lysosomal-mediated cell death). Programmed cell death (PCD) is referred to apoptosis, autophagy, and programmed necrosis, that all these three pathways can determine the fate of malignant neoplastic cells alongside each other. It gets more complicated when a housekeeping process known as autophagy, that regulates physiologic roles, is also able to expand the cancer cell survival (2).

Autophagy as the second type of programmed cell death (PCD type II) (3) is evolutionally conserved and a catabolic procedure (4) that begins with forming autophagosomes, consisting of a two-membrane structure covering cytoplasmic harmful macromolecules and organelles, and ultimately ends up with recycling trapped components (5, 6).

In general, autophagy plays a fatal role in cell homeostasis by inducing pro-survival signals, that are required during starvation periods and stresses such as growth factor deprivation (7).

Autophagy pathway consists of following phases: induction, autophagosome nucleation, elongation and completion, lysosome conjunction, and degradation (8).

**Table 1:** Profile of specifications of patients with de novo AML

**Specification **	**%of patient samples**
**Sex**	
Male	44
Female	56
**Age(years)**	
Median	(45)
Range	(1-89)
0-10	10
10-70	70
70<	20
**AML (M3,non-M3)**	
AML M3	36
AML non-M3	64
**Specimen type**	
BM	82
PB	18
**Blast**	
Median	(80)
Range	(20-89)

**Figure 1:**
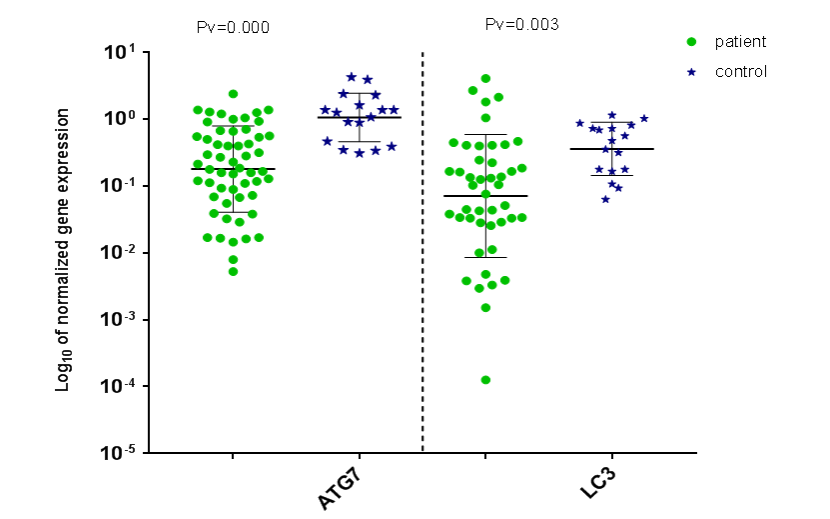
Distribution of normalized gene expression level of ATG7 (left) and LC3 (right) for AML patient and control samples

**Figure 2:**
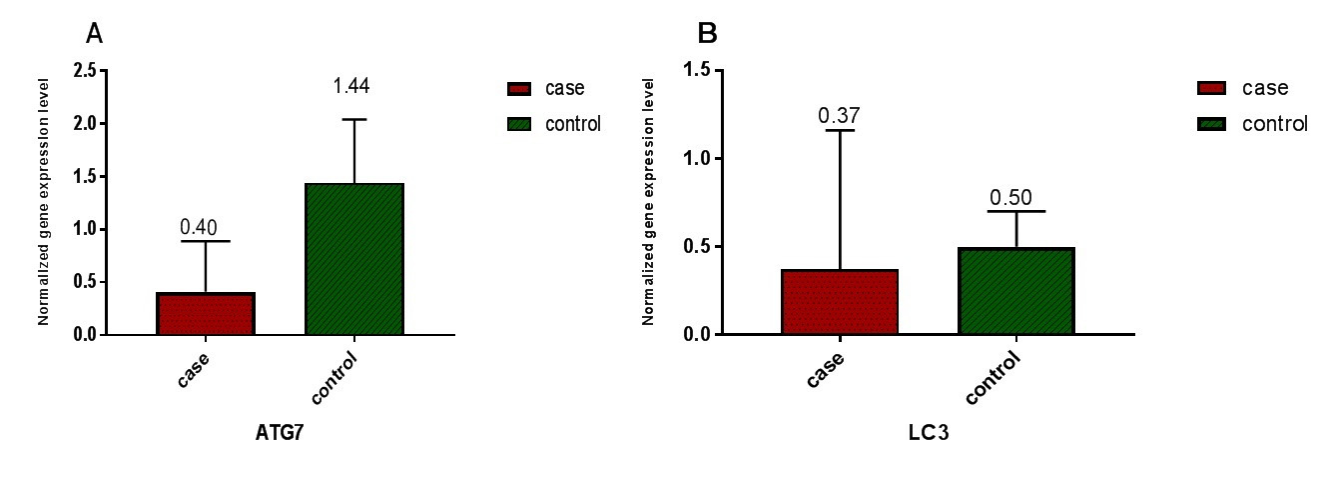
Relative expression of ATG7 and LC3 in 55 AML patients and 17 healthy volunteers was measured based on CT values and normalization against reference gene (ABL). A) A significant difference (*P < *0.0001) in ATG7 gene expression between AML patients and healthy volunteers was detected. Relative ATG7 gene expression level of 0.409 ± 0.483 (SD) was measured in AML patients in comparison to 1.44 ± 0.9 (SD) in normal control group B) A significant difference (*P < *0.005) between LC3 gene expression in AML patients and healthy volunteers was detected. Relative LC3 gene expression level of 0.37 ± 0.79 (SD) was measured in AML patients in comparison to 0.50 ± 0.34 (SD) in the normal control group

**Figure 3:**
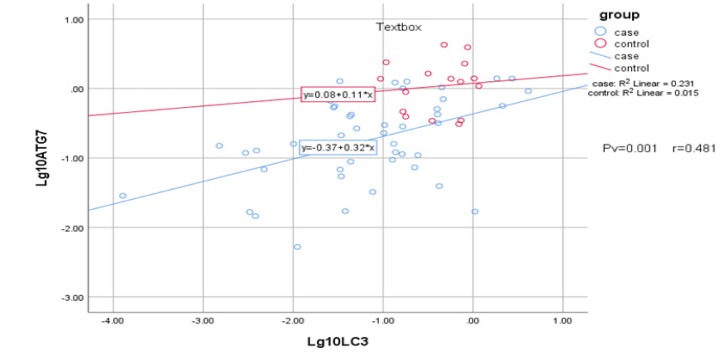
Statistical analysis by means of Pearson’s chi-squared test demonstrate a positive correlation between ATG7 and LC3 gene expression (*P *= 0.001, *r *= 0.481)

During nucleation, proteins and lipids are involved in phagosome membrane formation. Elongation and completion phase ends up with two ubiquitin-like conjugation systems. 

The first system involves ATG7 and ATG3 accounting for lipid modification in Light Chain 3 (LC3), altering inactive LC3I to active LC3II. LC3 plays its role in mounting the cargo (i.e. proteins and organelles) into the forming phagosome. The second system involves ATG7 and ATG10 accounting for ATG12-ATG5-ATG16L complex formation that is required for LC3 effective lipidation. Then, autophagosomes combine with lysosome to form autophagolysosome, a site for degradation of trapped components and then recycling begins (9).

Despite all, autophagy role in regulation between death and survival of cancer cells (10) has been yet in controversy that would be cleared when diverse outcomes of autophagy are separately studied in different tumor formation steps.

In recent years, several studies have focused on autophagy in acute leukemia cells, so in this study we assessed the relation between defect in autophagy and AML occurrence by assessment of autophagy key genes expression including ATG7 and LC3 in de novo AML patients.

According to these results the other angles of knowledge in AML pathogenesis would be expanded in order to discover novel specific ways in diagnosis, prognosis, targeted therapy, and monitoring.

## ٍExperimental

*Patients and Healthy Volunteers*

The samples were obtained from 55 AML patients in both sources of bone marrow (BM) and peripheral blood and 17 BM and PB samples of healthy volunteers from years 2016 to 2017 with receiving informed consent according to institutional guidelines. The patients were diagnosed at Taleghani Hospital, Tehran, Iran, as diagnosis was determined in accordance with the FAB classification system, which is based on morphology and defining specific immunophenotypes.

*RNA Isolation and cDNA Synthesis*

Total cellular RNA was extracted from BM using RNeasy Kit (Qiagen, Germany). Following the extraction, the integrity of RNA was measured by the NanoDrop (Thermo Scientific, Wilmington, North Carolina, USA). All samples included in the study showed high purity (OD 260/280 nm ratio *>*1.8).

Subsequently, 1 *μ*g of RNA was transcribed into cDNA to a final volume of 20 μL by means of cDNA Synthesis Kit (Thermo Scientific, Qiagen, Hudson, NH, USA). After synthesizing cDNA, an equal amount of cDNA from each sample of control and patient was used as substrate for qRT- PCR amplification.

*Real-Time PCR (qRT-PCR)*

The utilized primers in this study were designed via Oligo7.56 software and data were obtained from the NCBI Blast database. The primer sequences were as followed, for ATG7 forward primer (5-ATTGCTGCATCAAGAAACCC-3) and reverse primer (5-GATGGAGAGCTCCTCAGCA-3, for LC3 forward primer (5-CGTCCTGGACAAGACCAAGT-3) and reverse primer (5-CTCGTCTTTCTCCTGCTCGT-3), for ABL forward primer (5-AGTCTCAGGATGCAGGTGCT-3) and reverse primer (5-TAGGCTGGGGCTTTTTGTAA). ABL, as a housekeeping gene, was the control gene in this study. Using these primers allowed ATG7, LC3, and ABL cDNA to be specifically amplified. Consequently, ATG7, LC3, and ABL mRNA expression in patient and healthy volunteer samples were analyzed by qRT-PCR (Rotor Gene 6000, Bosch, Qiagen, Germany). The components in the qRT-PCR reaction for each target consisted of 1 μL of cDNA targeted template, 0.5 μL of each forward and reverse primer, 5 μL of RealQ Plus 2x Master Mix Green- Low ROX (Ampliqon, Denmark), and 3 μL depc-water for a total reaction volume of 10 μL. For each qRT-PCR reaction, a standard curve was provided, using five consecutive 1:20 dilutions of a positive sample (1, 0.1, 0.01 and 0.001) , as previously set up (11). The thermal cycling conditions for each reaction was as followed: 95 °C for 10 min as holding (ATG7, LC3 and ABL), 95 °C for 10 seconds as denaturation (ATG7, LC3 and ABL), 58.7 °C, 61.5 °C and 65 °C for 10 seconds for ATG7, LC3 and ABL, respectively, as annealing, 72 °C for 25 seconds as extension (ATG7, LC3 and ABL), in 40 cycles (denaturation, annealing and extension). The relative quantification of mRNA expression for each sample (fold change = FQ) was calculated using the Livak method (2^-∆∆ct^) (12).

*Statistical Analysis*

Data analysis was executed using the SPSS Statistics 16.0 and GraphPad Prism 6.07 software. In order to normalize distribution of data in SPSS, log10 was used in analysis. Applying both Shapiro- Wilk and Kolmogorov-Smirnov tests for log10ATG7 and log10LC3 proved normality of distribution in case/control, AML state as M3 and non-M3 AML, age and gender. In addition, *t*-test was used to determine any significant difference in ATG7 and LC3 expression between AML patients and normal control group. Also, Pearson’s chi-squared test was used to measure the linear correlation between ATG7 and LC3 expression.

A significance threshold level of *α *= 0.05 was applied, and the results are expressed as mean ± standard deviation.

## Results

*Profile of Patient Sample Specifications*

The analyzed samples in our study were obtained from 55 de novo AML patients, categorized in two groups of AML M3 and non-M3 AML, and 17non leukemia volunteers varying in gender and age (Table1).

*ATG7 and LC3 gene expression in AML and healthy samples*

ATG7 and LC3 gene expression were analyzed by real-time PCR in AML and healthy samples. For validation of the ∆∆CT method, the amplification efficiencies of reference and target were obtained, and they were approximately equal. In order to do so, the prepared cDNA was diluted over a 100-fold range. As the calibrator, the average expression of ATG7 and LC3 was obtained in 12 BM and 5 PB samples of healthy volunteers. The mean CT values (± SD) of ABL, ATG7, and LC3 were 26.03 ± 2.09, 25.94 ± 1.77 and 27.49 ± 2.17, respectively in the control samples. ABL, ATG7, and LC3 gene expression in AML samples were analyzed using the same technique. The results were as 25 ± 3.82, 27.48 ± 4.20, and 27.76 ± 3.49, respectively for ABL, ATG7, and LC3. Subsequently, the CT values obtained from ATG7 and LC3 were normalized against the internal reference gene, ABL, for both AML positive and normal control group samples (Distribution of normalized gene expression level of ATG7 and LC3 in AML patients and control samples is demonstrated in Figure1). Then, a statistical comparison was made between the normalized values of AML and normal control samples, which revealed a significant difference (*P* < 0.05) between AML and control samples for both ATG7 and LC3 gene expression (Figure 2A and B). The mean gene expression level (± SD) in AML and normal control samples for ATG7 was measured 0.409 ± 0.483, 1.44 ± 0.9, respectively and for LC3 was 0.37 ± 0.79 and 0.50 ± 0.34, respectively.

A gene expression level in the range of 95% confidence interval, defined based on the average gene expression level for ATG7 and LC3 in control population, was considered as 0.72–2.89 and 0.25–1.0, respectively. The results are as followed, 81.81% decrease and 18.18% equal to range for ATG7 gene expression and 75.55% decrease, 13.33% equal to range and 11.33% overexpression for LC3 gene expression in patients compared to control samples. Expression levels below the threshold of the intermediate ranges for ATG7 and LC3 were defined as low expression levels, 0.0052–0.72 and 0.0001–0.25 for ATG7 and LC3, respectively. Conversely, high expression levels are defined as 1.0-4.11 for LC3 gene.

*Correlation between ATG7 and LC3 Expression Levels*

Statistical analysis was applied to determine possible correlation between expression of ATG7 and LC3. The analysis showed positive and significant correlation between ATG7 and LC3 gene expression (Pv = 0.001 and r = 0.481) in both AML patients and the control samples, suggesting dependency in their expression (Figure 3). However, no significant relation was detected in age, gender, and AML state as M3 and non-M3 with interested genes, neither for AML nor control samples.

## Discussion

This is now obvious that cancer progression is related to autophagy, although its exact roles in different steps of cancer progression are not clearly known and it`s also in controversy for some conditions (13). Regarding the basic difference between tumorigenesis in solid tumors and leukemogenesis in hematopoietic malignancy, the studies deciphering the relation between acute leukemia and autophagy are interestingly growing. Therefore, we aim to find out mentioned relation in acute myelogenous leukemia (AML).

First observation to show a relation between autophagy and cancer was monoallelic deletion of BECN1 gene coding beclin1 in breast, ovary, and prostate cancers. Moreover, another mutations related to autophagy genes in other cancers such as gastric and colorectal were detected (13). Also, LC3 expression in radioresistant breast cancer cells is found intensively different compared to radiosensitive breast cancer cells, and autophagy is considered to be responsible for radioresistant breast cancer cells’ survival (14). As in a study in 2014, Clioquinol induced mTOR pathway suppression that resulted in autophagy-mediated apoptosis in leukemic and myeloma cells (15), also it was shown, by Dennis J. Goussetis in 2010, that Arsenic trioxide (A_2_O_3_) generates anti-leukemic responses in primary progenitors of AML by utilizing autophagy mechanisms *in-vitro* (16).

Our results reveal decrease in expression of LC3 and ATG7 genes for majority of de novo AML patients compared to control group, similar as results in study by As Watson in 2015 that showed decreased autophagy gene expression in human AML (MLL-ENL cell line) (17). So these findings provide possible evidence that the loss of autophagy genes may be generally beneficial for tumor growth, but there are also studies that don’t necessarily support this issue (18).

Therefore, autophagy expression changes can be valuable in diagnosis, prognosis, treatment targets, and disease monitoring (19-21). As we expected, our results mostly show decrease in autophagy gene expression. However, in few patients with gene overexpression and also in some others no significant changes in autophagy genes expression were detected (two parts of the results that in fact led us into new assumption). As we didn′t see similar gene expression levels in all AML patients, this suggests that Leukemogenesis is related to autophagy process in different ways and does not follow a specific pattern.

In many studies autophagy changes were observed in malignancies under chemotherapy and radiotherapy, and they concluded that autophagy genes were overexpressed and resulted in resistance to treatment, so attempted to attenuate autophagy for improvement in treatment outcome (18, 21, 22). Also, these studies were worked up on refractory patients, in spite of our study on de novo AML patients.

Based on our study, there was no significant correlation between age and these two interested genes expression or between gender and autophagy genes expression. Regarding the decrease in LC3 and ATG7 expression in AML patients compared to control samples and no significant correlation between expression of these genes in AML state as AML-M3 and non-M3 AML, that are naturally different, we suppose that autophagy changes are not directly involved in creating AML by their own, but weakened autophagy mechanisms can be in favor of leukemogenesis.

We conjecture that AML patients who indicated lower expression of autophagy genes during AML onset are as the same patients who are therapy sensitive, owing to their de novo weakened autophagy system; on the other hand those patients who indicated expression of autophagy genes the same as the control sample and also those with overexpressed autophagy genes at the AML onset are the same as the patients who indicate therapy resistance, owing to their intact autophagy system.

Based on our data, in contrast to the current belief that considers autophagy as a double-edged sword and efforts to find answers to the controversial sides of autophagy behaviors, we suppose that in fact fronting with two different faces of autophagy, one as a weakened housekeeping system on the onset of AML and the other face as the origin of resistance to therapeutic procedures, comes from two sides of autophagy in different patients. Actually, we suppose the former ends up with therapy sensitive patients and the latter initiates with the patients of normal or overexpressed autophagy genes. 

To prove such a claim another extended study is required to monitor the patients since AML onset till the therapeutic procedure and compare autophagy levels in these two phases of AML together, with the same patients. If such assumption was proved, in future we would be able to characterize our patients into two groups, one as weakened autophagy expression in AML onset accompanied with favor prognosis, and the other as normal or overexpressed autophagy accompanied with poor prognosis. By which we can hope to choose more suitable therapeutic policies in favor of patients.

## Conclusion

Although abundant efforts have been taken in AML diagnosis, prognosis and treatment, scant progressions has been achieved in recent decades. So, new approach to leukemogenesis and involved related pathways seems to be necessary. Presented results in this study indicate that autophagy genes expression in de novo AML patients are mostly decreased, although in previous studies in relapsed patients, gene overexpression has been detected. Therefore, more studies are required for enhanced utilization of this pathway in diagnosis, prognosis, and treatment.
